# Tumor cell p38 inhibition to overcome immunotherapy resistance

**DOI:** 10.21203/rs.3.rs-3183496/v1

**Published:** 2023-08-19

**Authors:** Jason J. Luke, Rebekah E. Dadey, Ryan C. Augustin, Sarah Newman, Krishna B. Singh, Rose Doerfler, Sarah Behr, Patrice Lee, Brian Isett, Christopher Deitrick, Aofei Li, Marion Joy, Carly Reeder, Katelyn Smith, Julie Urban, Lorenzo Sellitto, Mark Jelinek, Susan M. Christner, Jan H. Beumer, Liza C. Villaruz, Aditi Kulkarni, Diwakar Davar, Andrew S. Poklepovic, Yana Najjar, Dan P. Zandberg, Adam C. Soloff, Tullia C. Bruno, Lazar Vujanović, Heath D. Skinner, Robert L. Ferris, Riyue Bao

**Affiliations:** 1 Hillman Cancer Center, UPMC, Pittsburgh, PA, USA; 2 Department of Medicine, University of Pittsburgh, Pittsburgh, PA, USA; 3 Pfizer, Inc. Boulder, CO, USA; 4 Cancer Bioinformatics Core, UPMC, Pittsburgh, PA, USA; 5 Department of Pathology, University of Pittsburgh, Pittsburgh, PA, USA; 6 Translational Pathology Imaging Laboratory, UPMC, Pittsburgh, PA, USA; 7 Department of Immunology, University of Pittsburgh, Pittsburgh, PA, USA; 8 Department of Otolaryngology, University of Pittsburgh, Pittsburgh, PA, USA; 9 Department of Biostatistics, University of Pittsburgh, Pittsburgh, PA, USA; 10 Biostatistics Core, UPMC, Pittsburgh, PA, USA; 11 Cancer Therapeutics Program, UPMC Hillman Cancer Center, Pittsburgh, PA, USA; 12 Division of Hematology/Oncology, Department of Medicine, School of Medicine, University of Pittsburgh, Pittsburgh, PA, USA; 13 Department of Pharmaceutical Sciences, School of Pharmacy, Pittsburgh, PA, USA; 14 Departments of Massey Cancer Center, Virginia Commonwealth University, Richmond, Virginia, USA.; 15 Departments of Internal Medicine, Virginia Commonwealth University, Richmond, Virginia, USA.; 16 Department of Cardiothoracic Surgery, University of Pittsburgh, Pittsburgh, PA, USA; 17 Department of Radiation Oncology, University of Pittsburgh, Pittsburgh, PA, USA

**Keywords:** p38, MAPK, immune exclusion, tumor-intrinsic, tumor microenvironment, immunotherapy, non-T cell-inflamed tumors, resistance, response, clinical trial, human specimens

## Abstract

Patients with tumors that do not respond to immune-checkpoint inhibition often harbor a non-T cell-inflamed tumor microenvironment, characterized by the absence of IFN-γ-associated CD8^+^ T cell and dendritic cell activation. Understanding the molecular mechanisms underlying immune exclusion in non-responding patients may enable the development of novel combination therapies. p38 MAPK is a known regulator of dendritic and myeloid cells however a tumor-intrinsic immunomodulatory role has not been previously described. Here we identify tumor cell p38 signaling as a therapeutic target to potentiate anti-tumor immunity and overcome resistance to immune-checkpoint inhibitors (ICI). Molecular analysis of tumor tissues from patients with human papillomavirus-negative head and neck squamous carcinoma reveals a p38-centered network enriched in non-T cell-inflamed tumors. Pan-cancer single-cell RNA analysis suggests that p38 activation may be an immune-exclusion mechanism across multiple tumor types. P38 knockdown in cancer cell lines increases T cell migration, and p38 inhibition plus ICI in preclinical models shows greater efficacy compared to monotherapies. In a clinical trial of patients refractory to PD1/L1 therapy, pexmetinib, a p38 inhibitor, plus nivolumab demonstrated deep and durable clinical responses. Targeting of p38 with anti-PD1 has the potential to induce the T cell-inflamed phenotype and overcome immunotherapy resistance.

Immunotherapy with immune-checkpoint inhibition (ICI) has improved outcomes for patients with cancer however most do not benefit. The T cell-inflamed tumor microenvironment (TME), characterized by CD8^+^ T cell infiltration, type I/II interferon (IFN) gene expression and antigen-presentation machinery^1,2^, is an important cancer immunotherapy biomarker^3^ that can also facilitate the identification of molecular correlates of immune exclusion^4^. Wnt/β-catenin signaling was the first described tumor-intrinsic mechanism of immune exclusion, initially in melanoma^5^ then across tumors^6^. We described a molecular atlas of tumor-intrinsic mechanisms of immune exclusion across cancer types using bulk RNA sequencing (RNAseq) confirming our previous findings and nominating further potential therapeutic targets^7^. This previous work did not incorporate clinical biomarker stratification and we hypothesize that refinement of this work within tumor types may enhance patient selection for immune-exclusion targeted therapeutic approaches. p38 mitogen-activated protein kinases (MAPK) are stress mediators strongly associated with progression of several tumor types, with known roles regulating proliferation and senescence as well as apoptosis^8,9^. Within the immune compartment, p38 induces proinflammatory and immunosuppressive myeloid cells and cytokines as well as regulates dendritic cell maturation, type I interferon responses and antigen presentation^10,11^. Within stromal cells of the TME, p38 has also been identified to suppress adaptive immune responses^12^.

To further understand molecular correlates of immune exclusion within clinically relevant biomarker defined populations, we investigated head and neck squamous cell carcinoma (HNSCC) where stratification of care by the presence of the human papillomavirus (HPV) dictates treatment. We performed an initial analysis of RNAseq data from 481 tumors from The Cancer Genome Atlas (TCGA) HNSCC cohort^13^, in concert with pathway analysis, to identify oncogenic pathways differentially activated in non-T cell-inflamed tumors. Given the etiologic impact of HPV in HNSCC, we focused on the 395 HPV-negative tumors (Fig. 1a). Using a T cell-inflamed gene expression signature^6,7,14,15^, we observed 36% versus 30% of tumors expressing the T cell-inflamed versus non-inflamed phenotype, respectively. We detected 67 pathways significantly activated in non- inflamed relative to inflamed tumors (z-score≥1.95, *P*<0.05) from causal network analysis^16^ (Fig. 1a). Functional annotation of the pathways revealed two main themes, with 43 pathways representing the MAPK, HIF-1, and T cell-receptor regulation of apoptosis, and 24 representing IL6 pathway and oxidative stress (Fig. 1b).

Our previous pan-cancer analysis demonstrated that tumors with reduced T-cell inflamed gene expression showed higher numbers of co-occurred activated pathways^7^. We observed a similar trend in HPV-negative HNSCC, where pathway score was significantly and inversely correlated with T cell-inflamed gene expression (R^2^=0.726, *P*<0.05) (Fig. 1c). Of 67 pathways, 59 were independently validated in an oral cancer cohort from The International Cancer Genome Consortium^17^ (ICGC-ORCA; *P*<0.05) (Fig. 1d). Twenty-four programs were predicted to be activated by transcriptional factors such as HIF1A, the master regulator of cellular response to hypoxia, whereas the other 35 were driven by kinases, hormones, or other non-transcriptional factors (Fig. 1d). Consistent with our prior work^18–20^, CTNNB1 was strongly associated with non-T cell-inflamed tumors. In addition, we observed p38 (*MAPK14*), as a drug target where clinical therapeutics have been previously developed that was also strongly associated with the non-T cell-inflamed phenotype. Multiple molecular targets were identified with associated drugs (**Table S1**). We chose to focus specifically on p38 given that our research group had immediate access to pexmetinib, a p38 inhibitor, for re-purposing in immunotherapy combination clinical trials.

To investigate the cellular source of these immune exclusion signaling pathways, we analyzed two HPV-negative HNSCC single-cell(sc) RNAseq datasets (cohort A: Puram et al.^21^; cohort B: Kurten et al.^22^). We started with the gene expression sparse matrix (log_2_(TPM/10 + 1) in cohort A, log normalized Unique Molecular Identifier (UMI) counts in cohort B, cell annotation, and malignant cell labels publicly available from both cohorts, and selected primary tumors of advanced stage (T3/4) from HPV-negative patients for our analysis. After filtering, cohort A^21^ consisted of 2435 cells from tumor specimens of 11 patients, with UMAP showing distinct populations including epithelial cells, fibroblasts, T cells, B cells, and macrophages (Fig. 2a). For each pathway, we computed an expression score in individual cells by averaging the levels of all downstream target molecules involved in a pathway taking into consideration the change of expression direction. Among the tumor, stromal, and immune cell populations, malignant epithelial cells showed the highest pathway scores (Fig. 2b). A previous study reported p38 activation in fibroblasts as driving absence of T cell infiltration in triple-negative breast cancer^12^. In our study, the tumor cell-expressing p38 pathway score was significantly and dominantly higher than that from fibroblasts and other cells, suggesting that tumor cells are the major contributor to the p38 pathway expression in TME (**Fig. S1**).

We then sought to understand differences in tumor cell-specific pathway expression for the low-T cell-infiltrated versus high-T cell-infiltrated groups. For initial clarity, we focused on the extreme phenotypes based on T cell fraction from lower to higher out of all cells sequenced in the TME. Out of 11 patients, five had tumors of at least 40 malignant epithelial cells (ranging 113– 330 per sample) and were included in further analysis. We detected elevated expression in 50 out of 59 pathways from tumors of lower levels of T cell infiltrate, with 49 showing significant changes at FDR 0.10 (Fig. 2c; **Table S2**). Tumor cell-expression of CTNNB1 and p38 pathways were significantly upregulated in low-T cell-infiltrated relative to high-T cell-infiltrated tumors (FDR-adjusted *P*=0.0435 and 0.0036, respectively) (Fig. 2d). We independently validated the single-cell findings in a separate cohort of HPV-negative HNSCC patients (cohort B^22^) and observed the same results (**Fig. S2a-S2d; Table S3**). For cohort B, we verified our scRNAseq-based low- vs high-T cell-infiltrated tumor group assignment using lymphocyte-based immune scores from pathology H&E images. A significant enrichment of pathway expression was detected only in tumor cells (FDR-adjusted *P*<0.10) from both HNSCC scRNAseq studies and dominantly in low-T cell-infiltrated tumors. Taken together, these scRNAseq results validate the previous bulk RNA sequencing data and support a tumor cell-intrinsic role for regulating immune exclusion.

To prioritize druggable targets for potential combination immunotherapy beyond those previously identified, we developed an accumulative scoring system to nominate candidates integrating both bulk tissue and single-cell data. From the 59 pathways, we computed a combined relative rank for each pathway as the geometric mean of three values: the relative rank in TCGA (z-score higher to lower), the relative rank in ICGC for anti-correlation with T cell-inflamed gene expression (coefficient high to low negative), and the relative rank in scRNAseq tumor cell-expressing pathway scores of low- vs high-T cell-infiltrated tumors (Fig. 2e, 2f; **Fig. S2e, S2f**; **Table S4**). With this approach we integrated results from TCGA, ICGC and both HNSCC scRNAseq cohorts in order to narrow to six pathways (Fig. 3a), noting that VEGFA, a known mediator of immune-checkpoint inhibitor (ICI) resistance^23^, is a downstream target shared by all pathways (Fig. 3a). Further, these pathways form a core biomolecular protein-protein interaction network in non-T cell-inflamed tumors made up of WNT/β-catenin, VEGFA, and others centered at p38 (NFE2L2 [*alias* NRF2], CTNNB1, ESR1, EPAS1, MYC, etc.; Fig. 3b). We validated CTNNB1 and p38-activated tumors at the protein level in high-throughput tumor proteomics data of 101 HNSCC HPV-negative tumors from Clinical Proteomic Tumor Analysis Consortium^24^ (CPTAC). We observed an inverse correlation between CD8A protein abundance and CTNNB1 or p38 pathway target molecules (Spearman’s ρ=−0.47 and −0.27, respectively) (Fig. 3c, 3d). This inverse correlation was only detected in tumor cells (*P*<0.01) and not in adjacent normal tissue (*P*>0.25), further supporting that activation of CTNNB1 and p38 causally leads to immune exclusion in some tumors.

To quantitatively validate the spatial relationship of p38, CTNNB1, and CD8^+^ T cells, we performed immunohistochemistry (IHC) on 56 cores from tissue microarrays of oral cavity HNSCC. Sequential sections were co-registered to align the annotated tumor region of each core across slides, and the density of CTNNB1^+^ cells, phospho-p38^+^ cells, and CD8^+^ lymphocytes within the TME was quantified (**Table S5**). Phospho-p38^+^ cell density was positively correlated with CTNNB1^+^ cell density in tumor (Spearman’s correlation coefficient ρ=0.30, *P*=0.014; Fig. 3e), further supporting the co-activation of p38 and CTNNB1 pathways in HPV-negative HNSCC. We divided the tumor cores into four categories (phospho-p38_high_CTNNB1_high_, phospho-p38_low_CTNNB1_high_, phospho-p38_high_CTNNB1_low_, phospho-p38_low_CTNNB1_low_). In the context of CTNNB1-high tumor cores, we observed a significantly lower level of CD8^+^ tumor-infiltrating lymphocytes (TILs) in phospho-p38_high_CTNNB1_high_ versus phospho-p38_low_CTNNB1_high_ groups (*P*=0.038) (Fig. 3f).

We further investigated the spatial relationship of CTNNB1, p38, and CD8^+^ TILs using two panels of multispectral immunofluorescence (mIF) imaging with PhenoImager^™^ HT (Akoya Biosciences) on an additional cohort of 154 HN oral cavity cores (105 primary tumors and 49 normal mucosa; Panel 1: PanCK, CD3, CD8, PD1, phospho-p38 plus DAPI, and Panel 2: PanCK, CD68, CD163, CD11c plus DAPI). Tumor-infiltrating CD3^+^CD8^+^ T cells, particularly the CD3^+^CD8^+^PD1^+^ subset, were significantly depleted in tumors with high phospho-p38 relative to those with low phospho-p38 (Fig. 3g; Fig. 3h, left and middle panels; **Table S6**). Similarly, CD68^−^CD163^−^CD11c^+^ dendritic cells (DCs) were significantly lower in low vs high phospho-p38 tumors (Fig. 3h, right panel). Phospho-p38^+^PanCK^+^ cell density was significantly and inversely correlated with CD3^+^CD8^+^ T cells (Spearman’s ρ=−0.43), CD3^+^CD8^+^PD1^+^ T cells (ρ=−0.23), and CD68^−^CD163^−^CD11c^+^ DCs (ρ=−0.37), a pattern only detected in tumors but not in normal mucosa (*P*<0.05; Fig. 3i–k). In cases where multiple cores were derived from the same patient, we repeated the analysis after averaging cell densities into patient level, and the inverse correlation remained significant for tumor specimens (ρ=−0.57, −0.57, and −0.45, respectively; *P*<0.05).

Our prior work demonstrated CTNNB1 activation as driving the absence of T cell infiltrates in multiple tumor types^5,6^ and we were interested in investigating whether p38 presents a similar mechanism of immune exclusion across human solid tumors. We generated a 12-gene p38 pathway activation signature by an integrative analysis of multi-omics data from TCGA, ICGC, scRNAseq, and CPTAC (*ARG2, CD55, CYP4F3, FST, GCLC, IL1A, MIF, PLA2G4A, PTGS2, S100A12, SLC6A2, VEGFA*), and analyzed scRNAseq cohorts of lung squamous carcinoma (LUSC)^25^, lung adenocarcinoma (LUAD)^26^, renal clear cell carcinoma (RCC)^27^ (**Fig. S2**), and skin cutaneous melanoma (SKCM)^28^ (**Fig. S4**). Comparing low- vs high-T cell-infiltrated tumors, we identified a significantly higher tumor cell-expressing p38 activation score in low-T cell-infiltrated tumor specimens from LUSC, LUAD, and RCC (*P*<0.05; **Fig. S3**), but not in the SKCM scRNAseq cohort (**Fig. S4**) suggesting that certain tumor cell-intrinsic signaling pathways may impact immunotherapy outcomes in different cancers.

To functionally examine the causal impact of tumor cell-expressing p38 on immune exclusion, we generated shRNA knockdown of *MAPK14* (encoding p38 alpha isoform) gene stable human cell lines from five different cancer types including, HPV-negative HNSCC (PCI13), LUSC (H1703 and H520), RCC (RCC4), Her2^+^ breast carcinoma (BRCA) (MCF-7), and bladder carcinoma (BLCA) (T24). Immunoblotting confirmed knockdown of p38 by the V3LHS_316966 clone of the p38 shRNA plasmid in three clones (V2LHS_113215; V2LHS_113220) from shRNA stably transfected human cancer cells versus empty vector (EV) transfected control cells (**Fig. S5a**). We used EV control and corresponding p38 shRNA stably transfected (clone: V3LHS 316966) human cancer cells for the downstream experiments. To identify the effects of p38 knockdown in tumor cells on potential crosstalk between tumor and immune cells, we performed T cell migration assays using conditioned media from EV control and corresponding p38 shRNA human cancer cells. Conditioned media from the p38 shRNA human cancer cells attracted significantly more Jurkat T cells as compared to the conditioned media from the EV control cells (*P*<0.05; **Fig. S5b**).

The potential for pharmacologic inhibition of p38 MAPK to enhance ICI was explored using a small molecule p38 inhibitor (ARRY-614; pexmetinib) in combination with ICI in EMT6 and CT26 syngeneic murine models. BALB/c mice were injected with either breast mammary cell line EMT6 or colorectal carcinoma CT26. After four days of tumor growth, mice were treated with either anti-CTLA4/vehicle (200 micrograms, IP, on Days 1, 5, 8, 11 post initial growth phase) or anti-PD1/vehicle (200 micrograms, IP, on Days 1 and 15 post initial growth phase), with or without pexmetinib (30mg/kg every day for 21 days) (**Fig. S6a**). Mice treated with pexmetinib plus ICI demonstrated improved survival and reduced tumor growth compared to ICI or pexmetinib alone (**Fig. S6b-e**). These *in vivo* data as well as the totality of *in silico* and *in vitro* evidence nominates p38 inhibition as a high-priority therapeutic target to enhance ICI in patients with cancer.

To explore the safety and preliminary efficacy of p38 inhibition with ICI, we performed a phase I clinical trial of pexmetinib and nivolumab in study subjects with advanced solid tumors who had progressed on anti-PD1, with primary endpoints describing dose-limiting toxicity (DLT) and objective response per RECIST version 1.1. The baseline demographics for the subjects are shown in **Table S7**. Fifteen subjects were included in the study with 14 having at least one post-treatment radiographic evaluation and 13 evaluable for DLT. All were white, majority male (n=11/15; 73%) and seven > 65 years of age. Subjects dominantly demonstrated lactate dehydrogenase (LDH) above the upper limit of normal (n=11/15; 73%) and had elevated tumor burden defined as greater than five total cm by RECIST (n=13/15; 87%). Subjects had received up to five and a median of two prior lines of systemic therapy with all having a diagnosis of metastatic non-small cell lung cancer (NSCLC) or RCC except for one with esophageal cancer and one with choroidal melanoma.

Given that pexmetinib had previously undergone dose finding in myelodysplastic syndromes^29^, this study was initiated at the previously defined maximum tolerated dose of pexmetinib (800 mg PO daily for 30 days per cycle) with nivolumab 400 mg IV monthly. Using a logic-based Bayesian statistical model, pexmetinib dose was decreased throughout the study due to DLTs or inadequate safety profile before eventually identifying 200 mg as the recommended phase two dose of pexmetinib in combination with nivolumab (**Table S8**). Subjects received a median of 3 (range 1 to 26) cycles.

All subjects experienced at least one treatment-emergent AE (TEAE), with events occurring in > 50% of participants including hypoalbuminemia (10 participants, 67%), hyponatremia (10 participants, 67%), anemia (9 participants, 60%), hypocalcemia (9 participants, 60%), and rash (9 participants, 60%). The most common treatment-related adverse events include rash (13 participants, 87%), diarrhea (6 participants, 40%), AST elevation (6 participants, 40%), and ALT elevation (6 participants, 40%). (**Table S9**). Immune-related adverse events (irAEs) of clinical significance including but not limited to colitis or hepatitis were observed in most subjects who obtained clinical benefit for more than 6 months (n=5/6; 83%). These were managed per standard algorithms with steroids. These irAEs were observed to be relapsing in some subjects with pexmetinib start/stop and some subjects having multiple distinct episode irAEs (i.e. – transaminitis then gastritis etc).

Pharmacokinetic data was available for all subjects (**Tables S10** and **S11; Fig. S7a-c**). As pexmetinib dose increased, exposure increased with dose (**Fig. S8a-c**), half-life appeared to be dose-dependent, yet dose-normalized C_max_ and total clearance were not impacted (**Fig. S8d-f**). In line with the short half-life, there was no accumulation with continued dosing, and exposure was similar on days 29 and 1. AR00451575 metabolite exposure was a quarter to a third that of the parent pexmetinib, and the half-life appeared slightly longer. The metabolic ratio suggested a possible decreasing trend with dose (**Fig. S9a-b**, *P*=0.238 and 0.043 for AUC and C_max_, respectively). Evaluation of pexmetinib exposure by occurrence of DLT suggested that higher AUC may be (*P*=0.058) associated with DLT (**Fig. S9c-d**).

All but one subject had been previously treated with anti-PD1. All PD1 previously treated subjects met the criteria on entry for the Society of Immunotherapy for Cancer definition of anti-PD1 resistance^30^. Of the 14 radiographically evaluable subjects, three (21.4%) experienced irRECIST confirmed response, including two (14.3%) having RECIST confirmed response. Six patients (42.9%) experienced clinical benefit (defined as not having progression and/or not receiving subsequent treatment beyond six months; **Table S7**, Fig. 4a). Out of the six patients, five (35.7%) had not transitioned to a new therapy within one year of starting on protocol. The one anti-PD1 treatment-naive patient had progression on first evaluation. Study subject level characteristics as well as tumor molecular and size details are described in **Table S7**. Best overall response (BOR) is shown in Fig. 4a and tumor change over time in Fig. 4b. The median duration of response was not reached. The two RECIST confirmed responding subjects (NSCLC and RCC) notably completed two years on treatment and remain progression free after discontinuing therapy with representative images shown in Fig. 4c. Fig. 4d shows no association of time on prior treatment and clinical benefit on this study. Progression-free and overall survival are shown in **Fig. S10**.

We report a tumor-cell intrinsic role of p38 activation in driving immune exclusion using large-scale computational analysis with validation across *in vitro* and *in vivo* model systems. We demonstrate in a phase I clinical trial of subjects with PD1 refractory tumors that p38 inhibition with pexmetinib plus nivolumab is tolerable and can lead to deep and durable treatment responses. Our work warrants further mechanistic studies surrounding the impact of p38 across the TME and peripheral immune compartments in mediating immune exclusion. These data demonstrate that p38 is a novel therapeutic target for overcoming immunotherapy resistance and further clinical trials of p38 inhibition with ICI should be prioritized.

## Methods

### Human specimens.

Formalin-fixed, paraffin-embedded (FFPE) tumor tissue samples in HNSCC biobank were obtained from patients consented under The University of Pittsburgh institutional review board (IRB)-approved protocol (HCC#99–069). Blood samples were obtained from patients in phase Ib clinical trial NCT04074967 (n=15; 14 with RECIST v1.1 response outcome evaluable). The study protocol was approved by The University of Pittsburgh IRB-approved protocol (HCC#19–097). All samples have written-informed patient consent.

### Study cohort and datasets.

We collected HNSCC cohorts of multi-omics data from TCGA^13^ (*n*=481), ICGC^17^ (*n*=40), CPTAC^24^ (*n*=101), as well as pan-cancer single-cell sequencing data for the integrative analysis in this study. All data are from publicly available omics repositories including NCI’s Genomic Data Commons (GDC) and NCBI SRA.

### TCGA and ICGC analysis.

In TCGA cohort, samples were split into HPV-positive and HPV-negative subsets based on HPV status^13^. A predefined and validated 160-gene T cell-inflamed gene signature was utilized to categorize tumors into T cell-inflamed, intermediate, or non-T cell-inflamed using a two-tier scoring system following previous protocols established by our group^7^. In ICGC cohort, the T cell-inflamed gene expression was computed by averaging the normalized and log-transformed expression of the 160 genes in the signature and then correlated with each of the oncogenic pathway scores in a continuous manner.

### Drug-gene interaction.

Genes encoding upstream regulators of the 59 oncogenic pathways (e.g., *CTNNB1*, *MAPK14*, etc.) associated with immune exclusion were queried in The Drug Gene Interaction Database^31^ (DGIdb, http://www.dgidb.org) (v4.2.0, accessed June 21, 2023) for gene-drug interactions (inhibitor, activator, etc.) with default settings. The DGIdb integrates resources from 40 databases and consists of existing drugs including those that are FDA-approved, antineoplastic, and/or immunotherapies.

### Single-cell RNAseq analysis.

For the scRNAseq cancer cohorts, publicly available expression matrix, cell annotations, and malignant cell labels were downloaded and subject to downstream analysis. Within each cohort, the published data were already filtered and normalized from the study, hence no further quality processing steps were needed. For HNSCC cohorts, baseline tumors of HPV-negative and of advanced stage (T3/4) were used for analysis. HNSCC cohort A^21^ scRNAseq data were generated using SmartSeq2 and quantified in transcript-per-million (TPM) with log_2_(TPM/10+1) unit, and HNSCC cohort B^22^ scRNAseq data were generated using 10X Genomics 3’ V2 chemistry and quantified in UMI count matrix with log(UMI) unit. Within each dataset, after selecting the first 10 principal components using Principal Component Analysis (PCA), a neighborhood graph was computed based on the PCA representation of the data and visualized by Uniform Manifold Approximation and Projection (UMAP) dimensionality reduction technique with Leiden clustering. For the statistical comparison of tumor cell-expressing pathway scores in low- vs high-T cell-infiltrated tumors, data were further filtered to keep tumor samples of ≥40 malignant epithelial cells and ≥100 cells total within each dataset.

### Pan-cancer scRNAseq analysis of p38/MAPK signaling.

For each tumor, a p38-activation score was computed as the mean expression of the 12 genes involved in the signature identified in HPV-negative HNSCC, after scaling and centering across all tumor samples. These expression scores were then used to correlate with the T cell-inflamed gene expression across all tumors by Spearman’s correlation. Pathway activation scores were calculated for each individual tumor sample, requiring at least 50% of the cancer-specific target molecules to be upregulated (relative to its median expression across all samples from an individual cohort) and compared in the non-T cell-inflamed tumor group relative to T cell-inflamed.

### H&E and CD8 immunohistochemistry staining.

The hematoxylin and eosin (H&E) and CD8 IHC staining on HNSCC specimens was performed at UPMC Developmental Laboratory. Slides were cut at 4μm then baked for one hour at 60 degrees Celsius. The slides were cooled to room temperature then deparaffinized and hydrated in diH20. Slides were stained using Hematoxylin 560 MX (3801576, Eosin Phloxine 515 (3801606), Define MX-aq (3803598) and Blue Buffer 8 (3802918) (all catalog numbers from Leica Biosystems). The predilute CD8 (Leica Biosystems, PA0183) was stained on the Bond III instrument (Leica Biosystems) for 8 min. Prior to antibody placement, the antigen retrieval used was the ER2 (pH 8.9–9.1, Leica Biosystems, AR9640) for 20 minutes at 95 deg. Followed by the Bond Polymer Refine Detection Kit-DAB (Leica Biosystems, DS9800).

### CD8, CTNNB1, and phospho-p38 IHC quantification.

IHC TMA WSI core sections were de-arrayed, cells were segmented and DAB+ cells detected using QuPath^32^ (cell segmentation using medianRadiusMircons=1, sigmaMicrons=1.5, Hematoxylin threshold = 0.05; positive cell detection using DAB+ threshold = 0.2 nuclear DAB mean). Tumor regions were identified in H&E section of each core. All IHC core section images and cell detection objects were then registered to H&E cores using a rigid transform (wsireg https://github.com/NHPatterson/wsireg). Tumor regions were annotated on the H&E section of each tumor. By aligning these cell detections to H&E tumor area, IHC DAB+ cell density was measured inside and outside tumor region of registered cores as the percent of DAB+ / all cells detected for each corresponding IHC core section.

### Multispectral immunofluorescence staining and imaging.

Multispectral immunofluorescence staining was performed on 4um tumor FFPE sections: CD3 (cat# 85061S), CD8 (cat# ACI3160A), PD1 (cat# ab137132), PanCK (cat# sc-81714), phosphor-p38 (cat# 4511S), CD68 (cat# 76437S), CD163 (cat# CM353AK), CD11c (cat# 45581S), and DAPI. Automated staining of tissues was performed on the Leica Bond RX. For staining, Akoya Bioscience’s Opal 6-Plex Manual Detection Kit was used according to the manufacturer’s instructions (cat# NEL861001KT). Imaging was performed at 20X on the PhenoImager^™^ HT (Akoya Biosciences). InForm^®^ (v2.4.6) and Phenochart^™^ (v1.0) (Akoya Biosciences, Inc.) analysis software was used for whole slide scanning and regions of interest (ROI) spectral unmixing. Multi-channel composite TIFF files were exported for further data analysis.

### Multispectral immunofluorescence image analysis.

Cores were de-arrayed, scanned at 20X, and spectrally unmixed using InForm (Akoya BIosciences). Cell segmentation was performed on DAPI channel using Stardist^33^. Marker positive/negative cells were classified using a machine learning approach. Briefly, a small number of positive/negative cells (30~50 per class per ROI) were manually selected as the training set to build a Random Forests model, which was then used to predict all remaining cells. Measurement matrices consisting of centroid position (x,y), per-channel intensity, and class label of phenotyped cells were exported and further processed in R (v4.1.2).

### Cell Lines and culture condition.

Patient derived HPV-negative human squamous cell carcinoma of the head and neck cancer cells (HNSCC); PCI 13 and bladder carcinoma cell line T24 were a generous gift from Dr. Robert Ferris, human breast cancer cell line MCF-7 (ATCC HTB-22), human lung cancer cell lines H520 and H1703 from Dr. Laura A. Stabile, and human renal cell carcinoma cell line (RCC4) from Dr. Jodi K. Maranchie (UPMC Hillman Cancer Center, University of Pittsburgh, PA, USA). PCI 13 and T24 cells were maintained in IMDM medium supplemented with 10% FBS, 1% L-glut and 1% NEAA. RCC4 and MCF-7 cells were maintained in DMEM medium and 10% FBS, whereas H520s and H1703s cells were maintained in RPMI with 10% FBS. These cell lines were last authenticated by us in January of 2023. All cell lines were found to be free from mycoplasma and authenticated stocks of cell lines were used for this study. Empty vector transfected control cells or human MAPK14 (p38) shRNA knockdown cells were generated by stable transfection with EV control (GIPZ lentiviral empty vector shRNA control) or with three different clones of MAPK14 (p38) GIPZ lentiviral shRNA (clones Id; V2LHS_113220; V2LHS_113215; V3LHS_316966) lentiviral plasmids, respectively, and selected by puromycin (0.5 to 2.0 μg/mL) antibiotic. Glycerol stocks of GIPZ Lentiviral Empty vector shRNA control (Catalog No: RHS4349) and three different GIPZ Human MAPK14 shRNA plasmids (1- Catalog No: RHS4430–200212538: Clone Id: V2LHS_113215; 2- Catalog No: RHS4430–200183464; Clone Id: V2LHS_113220; 3 - Catalog No: RHS4430–200266677: Clone ID: V3LHS 316966) were purchased from “Horizon Discovery”.

### p38 protein Western Blot.

Cells will be washed twice with ice-cold PBS, lysed on ice in cell lysis buffer (RIPA buffer). The cell lysate will be cleared by centrifugation at 14 000 g for 20 minutes and protein content was quantified using Bradford method and equal amount of protein was subjected to sodium dodecyl sulfate-polyacrylamide gel electrophoresis. Proteins were transferred onto polyvinylidene fluoride membrane. After blocking with 5% non-fat dry milk in Tris-buffered saline containing 0.05% Tween-20, the membrane was incubated with the p38 (1:20,000 dilution; Cell Signaling Technology: catalog number # 9212) primary antibody for overnight at 4°C. Subsequently, the membrane was incubated with the appropriate secondary antibody, and the immunoreactive protein bands were visualized using chemiluminescence method. Each membrane was stripped and re-probed with housekeeping protein β- Actin (1:100,000 dilution; Sigma Aldrich: catalog number # A5441) to correct for differences in protein loading. Change in protein level was quantified using densitometric scanning of the immunoreactive band and corrected for β - Actin loading control by UN – SCAN - IT software (Silk Scientific).

### Preparation of tumor conditioned media.

1 ×10^6^ EV control and p38 shRNA stably transfected human cancer cells were plated in triplicate in 60 mm dish containing 10 % FBS and incubated for 48 hours. After incubation media was collected and centrifuged at 500×g for 5 minutes and medium supernatant was collected to use as a conditioned medium and then stored at −80 °C for downstream experiments.

### T-cell migration assay.

Jurkat T cells were cultured overnight in T cell migration medium (RPMI containing 1% FBS, 10mM HEPES buffer, pH 6.9). After overnight incubation 5×10^5^ Jurkat T cells were resuspended in 100 μl cell migration medium and were placed in upper chamber of 96-well transwell plate with 5-μm pore polycarbonate membrane (Corning). Conditioned medium from the EV control, p38 shRNA transfected cells and serum free media used as negative control were placed in the lower chamber of the plate. T cells were allowed to migrate into the lower chamber for 4 hours at 37 °C in CO_2_ incubator. The number of cells migrating to the lower chamber were counted manually by trypan blue using hemocytometer.

### Murine models and experiments.

The CT26 and EMT cell lines were obtained from the American Type Culture Collection (ATCC) and cultured according to ATCC guidelines. Four to six week old male and female BALB/c mice were injected with 5.0 × 10^5^ EMT6 cells or 1.0 × 10^5^ CT26 subcutaneously (s.c.). Tumors were measured every 2–3 d with a digital caliper in two dimensions (width and length) and presented as tumor volume (defined as 0.5 x *w*^*2*^ × *l*). Mice were monitored for survival up to 80 days after initial tumor inoculation. Tumors grew in an initial growth phase until palpable at 4 days and then mice were treated with 200 micrograms of anti-PD1/vehicle, IP, on Day 1 and 15 post initial tumor growth phase, or 200 micrograms of anti-CTLA4/vehicle, IP, on Day 1, 5, 8, and 11 post initial tumor growth phase. Mice receiving pexmetinib were treated with 30mg/kg inhibitor, orally, once daily for 21 days post initial tumor growth phase.

### Histopathologic analysis.

For histological analysis 4mm pieces of tumor tissue were fixed in either 10% formalin for H&E staining or Carnoy’s fixative for Periodic-acid Schiff (PAS) staining. Sectioning and staining were performed at UPMC Molecular Pathology Developmental Laboratory. All sections were reviewed by a pathologist in a blinded fashion.

### Clinical trial description and study subjects.

A Phase 1 investigator-initiated, open-label, multicenter clinical trial was conducted with written informed consent of the study subjects and otherwise in accordance with the provisions of the Declaration of Helsinki and the International Conference on Harmonization Guidelines for Good Clinical Practice (NCT04074967). Study subjects signed informed consent forms approved by site-specific ethics committees prior to undergoing study-related procedures. Study objectives were to establish safety and tolerability of pexmetinib with nivolumab and to select a recommended phase two dose of pexmetinib in combination with nivolumab in subjects with advanced solid tumors as well as to estimate the probability of objective response of pexmetinib in combination with nivolumab based on Response Criteria in Solid Tumors, version 1.1 (RECIST v1.1). Subjects received pexmetinib oral daily between 800–200 mg with nivolumab 400 mg IV monthly. Secondary objectives were to estimate the probability of toxicity of pexmetinib in combination with nivolumab, to estimate the overall survival (OS), progression-free survival (PFS), distribution of best clinical response and duration of response of subjects treated with pexmetinib in combination with nivolumab and to describe the preliminary anti-tumor activity of pexmetinib in combination with nivolumab based on immune-related response criteria (irRECIST).

### Clinical trial eligibility.

Eligible adult study participants had histologically confirmed stage III/IV advanced/metastatic solid tumors who lacked curative measures and for whom nivolumab was available. Subjects must have previous disease progression on anti-PD1 and RECIST measurable disease. Eligibility required an Eastern Cooperative Oncology Group performance status of 0–1, adequate cardiac function, and laboratory values within protocol-specified ranges. See the protocol document for further eligibility criteria.

### Clinical trial drug substance.

Pexmetinib was supplied as a formulated capsule, semi-solid suspension of micronized ARRY-614 hydrochloric acid (HCl) salt drug substance in Vitamin E d-alpha tocopheryl polyethylene glycol 1000 succinate (TPGS), Labrafac Lipophile WL 1349 and butylated hydroxytoluene (BHT). Pexmetinib formulated capsule drug product (micronized ARRY-614 HCl salt drug substance in a semi-solid carrier) was supplied in a 200 mg strength in a white opaque size “00” hard gelatin capsule. Nivolumab was sourced per clinical standard of care.

### Clinical trial efficacy assessments.

Disease status was assessed evaluated using RECIST ^34^as well as irRECIST changes per investigator assessment.

### Clinical trial pharmacokinetics.

Study subjects had blood samples collected for pharmacokinetic (PK) analysis of pexmetinib (ARRY-614) and its metabolite AR00451575 in EDTA tubes prior to and at 0.5, 1, 1.5, 2, 3, 4, 6, and 24 h after dosing on days 1 and 29, as well as prior to dosing on days 15, 43, and 57. Each blood sample was centrifuged at approximately 1,000 × g, and plasma was stored at −70 °C or colder until analysis. Plasma pharmacokinetic parameters were derived from the data by non-compartmental methods with WinNonlin (Certara, Princeton, NJ). Statistical analyses for pharmacokinetic parameter values were performed using SPSS 27.0 for Windows (SPSS Inc., Chicago, IL).

All samples were analyzed for pexmetinib and AR00451575 concentrations with an LC-MS/MS assay validated to FDA guidance over the range of 5–5,000 ng/mL.ARRY-614 (pexmetinib), AR00456800, AR00451575, and AR00441511 were provided by WuXi Apptec, Shanghai 200131, China PF numbers PF-06800302, PF-07315937, PF-07315936 and PF-07315935 respectively. Acetonitrile and water (both HPLC grade) were purchased from Honeywell (Charlotte, NC, USA 28105). Formic acid and DMSO were purchased from (Honeywell) FischerScientific, (Fairlawn, NJ USA 07410). Control EDTA human plasma was purchased from Lampire Biological Laboratories (Pipersville, PA, USA). Nitrogen for mass spectrometric applications was purified with a Nitrogen Generator (Parker Balston, Haverhill, MA, USA).

The LC system consisted of an Agilent (Palo Alto, CA, USA) 1200 thermostatted autosampler and 1200 Binary Pump, thermostatted column compartment (Agilent, Palo Alto, CA 94036). The column used was Synergi Polar-RP (4 μm, 100 × 2 mm). The autosampler temperature was also kept at 10 °C. Mobile phase solvent A consisted of 0.1% Formic acid and water. The mobile phase solvent B consisted of 0.1% Formic acid and Acetonitrile. A flow rate of 0.4 mL/min was maintained for 3 min and raised to 0.6 mL/min for 4 min. The initial mobile phase composition was 50% solvent A and decreased to 20% over 3.0 min, with the flow rate at 400 μL/min. At 3.1 min Solvent A was held at 20% until 4.0 min., with the flow rate increased to 600 μL/min. Between 4.0 and 4.1 min, solvent A was increased to 50% and held until 7 min with the flow rate of 600 μL/min followed by injection of the next sample. The total run time was 7 min. The injection volume was 5 μL. The approximate retention time of pexmetinib was 2.7 min, with a corresponding capacity factory of 2.14 with a void time of 0.86 min. The approximate retention time of AR00451575 was 1.6 min, with a corresponding capacity factory of 1.00, with a void time of 0.81 min.

Mass spectrometric detection was carried out using an ABI SCIEX (Concord, ON, Canada) 4000Q hybrid linear ion trap tandem mass spectrometer with electrospray ionization in positive multiple reaction monitoring (MRM) mode. The settings of the mass spectrometer for all analytes were as follows: curtain gas 40, CAD 4 Ion transfer voltage 5000 V, probe temperature 500 °C, GS1 40, GS2 40, declustering potential 40 V, entrance potential 10 V collision energy 40 V, collision cell exit potential 15 V, and dwell time 0.1 s. The MRM *m/z* transitions monitored were: 557.3>256.4 for pexmetinib; 561.3>256.3 for AR00456800 (614-IS); 573.3>272.3 for AR00451575 and 277.3>272.4 for AR00441511 (575-IS). The LC system and mass spectrometer were operated as previously reported with 1/y^2^ weighted linear regression^35^.

For the preparation of calibration standards and quality control samples, stock solutions of 1 mg/mL of pexmetinib, AR00451575, AR00456800 and AR00441511 were prepared in DMSO respectively. Pexmetinib and AR00451575 stocks were diluted together to prepare a working mix stock solution of 100 μg/mL in acetonitrile. AR00456800 and AR00441511 were diluted together to prepare a working IS mix stock solution of 100 μg/mL in acetonitrile. On the day of the assay, the 100 μg/mL pexmetinib and AR00451575 mix stock was diluted in steps of 10-fold in acetonitrile to obtain mixture working stocks, which were diluted in human plasma to produce a calibration range for both pexmetinib and AR00451575 of 5–5000 ng/mL. Quality control (QC) samples were prepared at QC Low (QCL) 15 ng/mL; QC Mid (QCM) 150 ng/mL; and QC High (QCH) 4000 ng/mL.

For sample preparation, a volume of 50 μL of the standard, QC, or sample plasma was pipetted into a microfuge tube and 10 μL of IS mix was added to each. Next, 200 μL of acetonitrile was added followed by vortexing for 1 min. Samples were centrifuged at 17,200 × g at room temperature for 10 min. Supernatants were transferred to autosampler vials, followed by injection of 5 μL into the LC-MS/MS system.

Regarding assay performance, QC based accuracies (N=6, each of 3 days) were 102.6–105.9% for pexmetinib and 99.4–105.9% for AR00451575. The intra- and inter-assay precisions were <6.7% and <7.4%, respectively for pexmetinib and <6.0% and <6.4% respectively for AR00451575. The stability of pexmetinib and AR00451575 in plasma at −80 °C was determined by assaying samples before and after storage for 30 months performed in replicates of four at the QCL, QCM and QCH concentrations. Stability of pexmetinib and AR00451575 in reconstituted samples in the autosampler for 72 h was pexmetinib 98.8–101.7% and AR00451575 98.2–98.9%. Incurred sample reanalysis of pexmetinib and AR00451575 samples respectively yielded the following results (%samples with a difference larger than 20% / median difference / median absolute difference): pexmetinib 13.1% / −6.0% / 7.5% and AR00451575 9.8% /−6.3% / 7.1%.

### Survival analysis.

The OS and PFS of patients were assessed using Kaplan-Meier analysis with observations right-censored.

### Statistical analysis.

The Wilcoxon signed-rank test was used to compare expression values between tumor groups. Differential gene expression analysis between groups was performed using empirical Bayes regression models in limma voom with precision weights. For multiple comparisons, p-value was adjusted using Benjamini-Hochberg (BH) FDR correction for multiple testing. Pearson’s correlation coefficient *r* was used for measuring statistical dependence between normalized and log_2_-transformed expression level of different genes, and between gene expression of the T cell-inflamed signature and pathways. scRNAseq tumor cell expressing pathway score comparisons between tumor groups were performed using linear mixed-effects models in lme4, with patient id as random effect and group of interest as fixed effect (formula: pathway_score ~ 0 + tumor_group + (1 | tumor_name), RMEL=FALSE). scRNAseq pathway score comparisons between malignant epithelial cells and fibroblasts were performed using linear mixed-effects models with a nested design (formula: pathway_score ~ 0 + cell_type + (1 | tumor_name/cell_type), RMEL=FALSE). REML was set to FALSE to use maximum likelihood for model fitting, and likelihood ratio test (LRT) was used to compute p-values. *P*<0.10 was considered statistically significant. All tests are two-sided unless otherwise noted. Statistical analysis was performed using R (v4.1.2) and Bioconductor (release 3.14).

For the clinical trial, safety analyses were performed on participants who received at least one dose of pexmetinib or nivolumab. Efficacy analyses were performed on treated participants with an on-treatment response assessment, including those who discontinued for progressive disease prior to the first scheduled response assessment. Adverse events were coded using the Medical Dictionary for Regulatory Activities (MedDRA^®^), version 22.0, and the severity of AEs and laboratory abnormalities were graded using the National Cancer Institute Common Terminology Criteria for Adverse Events (NCI CTCAE), version 5.0 (US DHHS 2017).

## Figures and Tables

**Figure 1.**
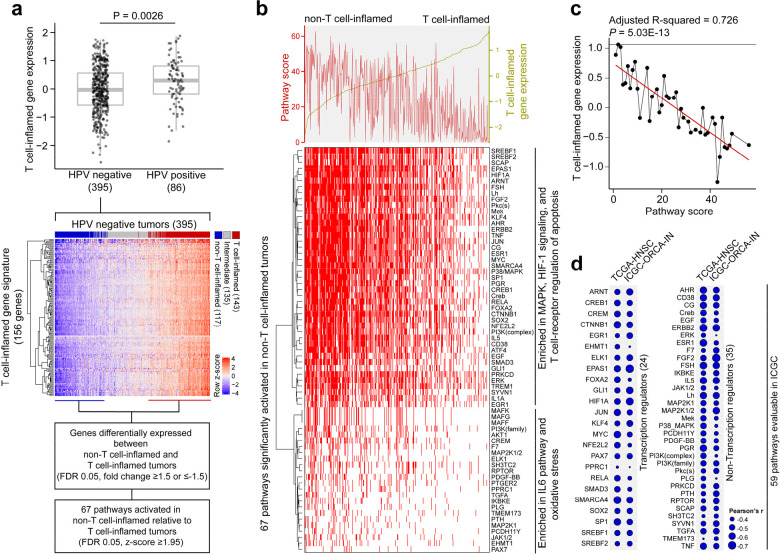
Activation of pathways associated with non-T cell-inflamed tumor microenvironment in HPV-negative head and neck squamous cell carcinoma. (**a**) Analysis workflow to identify pathway activation in non-T cell-inflamed relative to T cell-inflamed tumors using bulk RNAseq from TCGA. (**b**) Landscape of pathway activation in tumor (*n*=395) identified using causal network analysis. Pathway score was computed as the mean expression of all target molecules in the pathway. (**c**) Reduced T-cell inflamed gene expression in tumors correlates with a higher number of co-occurred activated pathways. (**d**) ICGC validation of activated pathways discovered in TCGA. Two-sided Welch Two-Sample *t*-test was used in comparing HPV groups (**a**, upper panel), limma voom with precision weights was used in identifying differentially expressed genes (**a**, first text box), linear regression was used in **c**, Pearson’s correlation was used in **d**.

**Figure 2.**
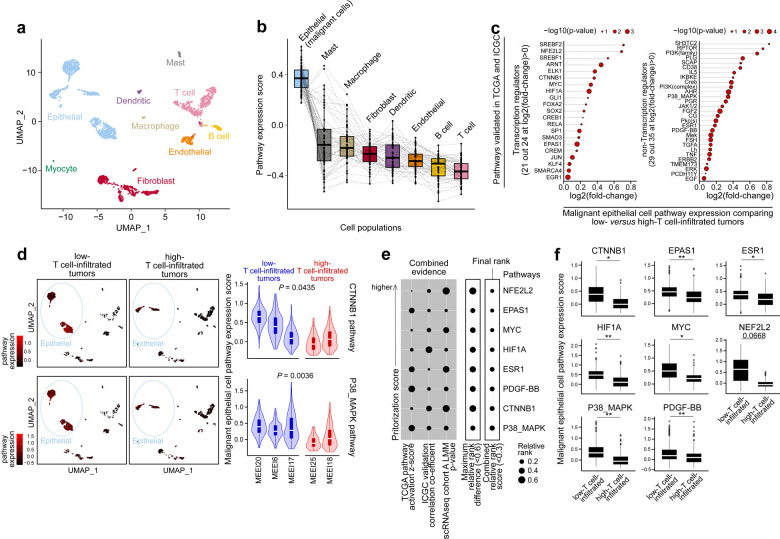
Immune-exclusion oncogenic pathways are activated in malignant cells from HPV-negative head and neck squamous cell carcinoma of low T cell infiltration from the Puram cohort. (**a**) Distribution of tumor, stroma, and immune cell subsets on UMAP (n= 2435 cells from 11 tumors). (**b**) Expression of 59 pathways from Fig. 1d across cell populations. Pathway scores were computed as the average expression of all genes involved in a pathway. (**c**) 50 out of 59 pathways showed higher expression in 774 malignant epithelial cells of low-T cell-filtrated relative to 253 malignant epithelial cells of high-T cell-infiltrated tumors. Bold font represents pathways at FDR 0.10. (**d**) Expression of CTNNB1 pathway and p38 pathway from low- vs high-T cell-infiltrated tumors. Five out of 11 tumors with at least 40 malignant epithelial cells per sample were included in analysis. (**e**) Eight pathways that passed prioritization score (combined relative rank) < 0.6 and maximum relative rank difference < 0.3. For each of the 59 pathways from Fig. 1d, its combined relative rank was computed as the geometric mean of three values: the relative rank in TCGA pathway activation (z-score higher to lower), the relative rank in ICGC anti-correlation with T cell-inflamed expression (coefficient highly to lowly negative), and the relative rank in scRNAseq pathway expression comparing malignant epithelial cells from low-T cell-infiltrated *versus* high-T cell-infiltrated tumors (p-values smaller to larger). (**f**) Expression of the eight pathways from **e**. Linear mixed-effects model via maximum likelihood was used in **c**, **d** and **f**, with tumor group as the fixed effect and patient id as the random effect. Likelihood ratio test (LRT) was used with the fitted model for computing p-values, followed by BH-FDR correction for multiple comparisons. Denotation: ** FDR-adjusted *P*<0.01, * FDR-adjusted *P*<0.05, otherwise the numbers are shown.

**Figure 3.**
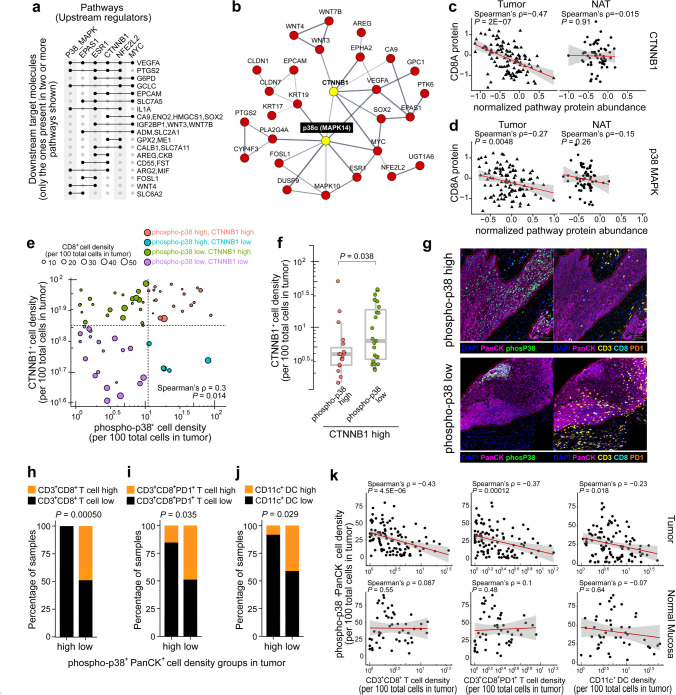
p38/MAPK pathway in non-T cell-inflamed tumors by proteomics and imaging experiments. (**a**) Six oncogenic pathways strongly associated with a non-T cell-inflamed TME and their shared downstream target molecules from curated literature in Ingenuity Knowledge Base. (**b**) Protein-protein functional interaction network based on the six pathways and shared downstream targets from **a**. Nodes with at least one connection are shown, from STRING functional protein association networks (confidence score >0.4; active interaction sources as “Experiments”, “Databases”, and “Co-expression”). (**c**) Proteomics correlation between CD8A protein abundance and p38 or CTNNB1 pathway proteomics score in HPV-negative HNSCC specimens from the CPTAC database. Tumors and adjacent normal tissues were shown. Pathway score was computed by averaging the normalized protein abundance of target molecules as defined in Ingenuity Knowledge Base. (**d**) IHC quantification of CTNNB1^+^, phospho-p38^+^, or CD8^+^ cell density in tumor. *n*=56 HNSCC cores are shown. Each datapoint represents one core, with color representing the four categories split by CTNNB1^+^ cell density high/low or phospho-p38^+^ cell density high/low, and size representing the CD8^+^ cell density. (**f**) Comparison of CD8^+^ cell density between the four specimen categories from **d**. (**g**) mIF imaging of phospho-p38^+^ high and low tumors showing the CD3^+^CD8^+^ and CD3^+^CD8^+^PD1^+^ TIL infiltrates in panCK^+^ tumor compartment. (**h**) Distribution of T cells and DCs in phospho-p38^+^ high and low tumors. *n*=154 cores were included in the analysis; this is an independent cohort of HNSCC specimens from what was shown in **e** and **f**. (**i**-**j**) Correlation between phospho-p38^+^PanCK^+^ cell density and (**i**) CD3^+^CD8^+^ T cell, (**j**) CD3^+^CD8^+^PD1^+^ T cell, and (**k**) DC density in normal mucosa (n=49 cores) or tumor (n=105 cores). Spearman’s correlation was used in **c**, **d,** and **i-k**. Two-sided Wilcoxon rank sum test was used in **f**. Two-sided Fisher’s exact test was used in **h**.

**Figure 4.**
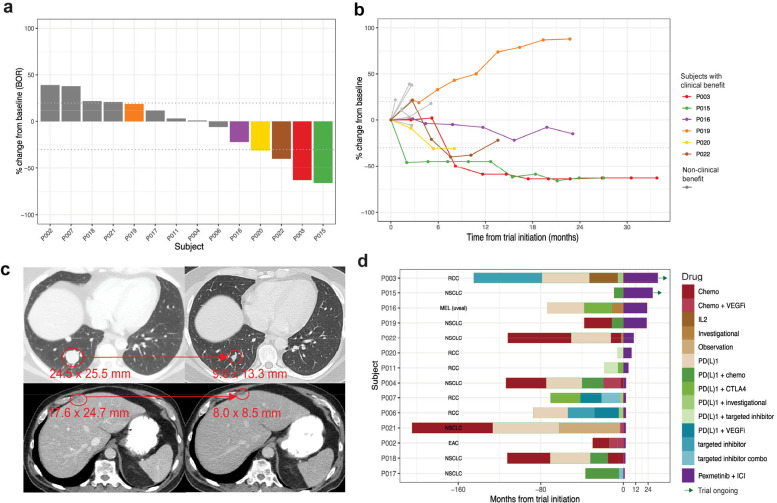
Pexmetinib plus nivolumab treatment overcomes resistance to ICI in patients with PD1 refractory tumors. *n*=14 radiographically evaluable patients shown. (**a**) Best overall response (BOR) per RECISTv1.1 or irRECIST as percent change from baseline. Colored subjects indicate those with >6 months clinical benefit. (**b**) Percent change from baseline through duration of clinical benefit for 14 patients. Colors indicate those with clinical benefit corresponding to the same subjects in **a**. Dashed lines in **a** and **b** indicate +20% (progression disease, PD) or −30% (partial response, PR) tumor size change. (**c**) CT scans from two patients experiencing response of approximately 50% (red arrows) in lesion shrinking. (top) renal carcinoma and (bottom) squamous NSCLC. Both subjects progressed on anti-PD1 in the prior line of therapy. Both subjects progressed on anti-PD1 in the prior line of therapy. Numbers in red indicate tumor size. (**d**) Swimmer’s plot denoting prior lines of therapy before trial initiation along with duration of clinical benefit on trial. RCC, renal cell carcinoma; NSCLC, non-small cell lung cancer; EAC, esophageal adenocarcinoma; VEGFi, vascular endothelial growth factor pathway inhibitor; ICI, immune-checkpoint inhibitor.

## Data Availability

The bulk and single-cell sequencing data are publicly available as follows: TCGA (Genomic Data Commons, https://portal.gdc.cancer.gov); ICGC (https://dcc.icgc.org); CPTAC (https://proteomic.datacommons.cancer.gov/pdc); HNSCC cohort A scRNAseq (*Puram et al.*, dbGap phs001474.v1.p1); HNSCC cohort B scRNAseq (*Kurten et al.*, NCBI PRJNA691564); LUSC, LUAD, RCC, SKCM scRNAseq (NCBI PRJNA622993, NCBI PRJNA591860, dbGaP phs002065.v1.p1, and NCBI GSE115978, respectively). The de-identified clinical data and processed biological data are provided in supplementary tables. Additional data are available from the corresponding authors upon request.
